# Supplementary material for: PFTK1 kinase regulates axogenesis during development via RhoA activation
Source: BMC Biol. 2023 Oct 31;21:240. doi: 10.1186/s12915-023-01732-w (PMC10617079; doi:10.1186/s12915-023-01732-w)
Supplement: Supplementary file 1 — Additional file 1: Fig. S1. Eip63E deficiency leads to concerted defects in axons and neurons of the Drosophila VNC. The combined penetrance of markers relevant to the same VNC structures are presented as one. Axon-associated penetrance reflects those calculated using HRP, BP-102 and Fasciclin-II antibodies. Neuron-related values include Neuroglian, Elav and Futsch-revealed penetrance. Penetrance was calculated as the % of defective embryos from the total embryos screened. Both deficient lines show the same nature of defects. Although it seems that defects associated to the Df(3L)E1 allele are more acute and appear later in development, Eip63E81 shows a robust axonal phenotype. ND: Not determined. Fig. S2. Eip63E functionally interacts with Rac1 and Cdc42 in D. melanogaster to regulate axogenesis. Ventral views of stage 14-15 whole mount embryos stained for BP102 antigen are shown head to the left. Panels show representative images for each specified genotype. An UAS-elavGal4 approach was used to drive Eip63E downregulation and either constitutively active or dominant negative forms of Rac1 or Cdc42 overexpression in neurons of independent D mel. lines. Fly genotype following crosses are indicated. (A-F) Neuronal functional interaction between Eip63E and Rac1. Only the combination of Eip63E deficiency and constitutively active Rac1 (UAS Rac1 V12; D), but not DN-Rac1 (UAS Rac1 N17; F), leads to an intermediate phenotype. (G-L) Functional interaction between Eip63E and Cdc42. Ventral views of the indicated genotypes of crosses between Eip63E and Cdc42 constitutive active form (UAS Cdc42 V12) or the dominant negative Cdc42 (UAS Cdc42 N17) are presented. Scale bar 50 μm. Fig. S3. Strategy for generation of PFTK1 KO murine line. The mouse was designed and developed by the Texas A&M Institute for Genomic Medicine (TIGM) (A) Strategy. Exon 6 (from nucleotide 18-Exon6 to nucleotide l-Exon7) of the Cdk.14 gene at chromosomes (4,803,391-5,380,19 7) was replaced for an IRES/bG [file 12915_2023_1732_MOESM1_ESM.pdf]

Stages	$Eip63E^{81}$ Axons	$Eip63E^{81}$ neurons	Df(3L)E1 Axons	Df(3L)E1 neurons
8-9	10.00	ND	ND	ND
10-11	35.84	36.10	7.14	28.60
12-13	50.00	52.86	88.00	85.70
14-15	85.71	44.50	96.67	100.00

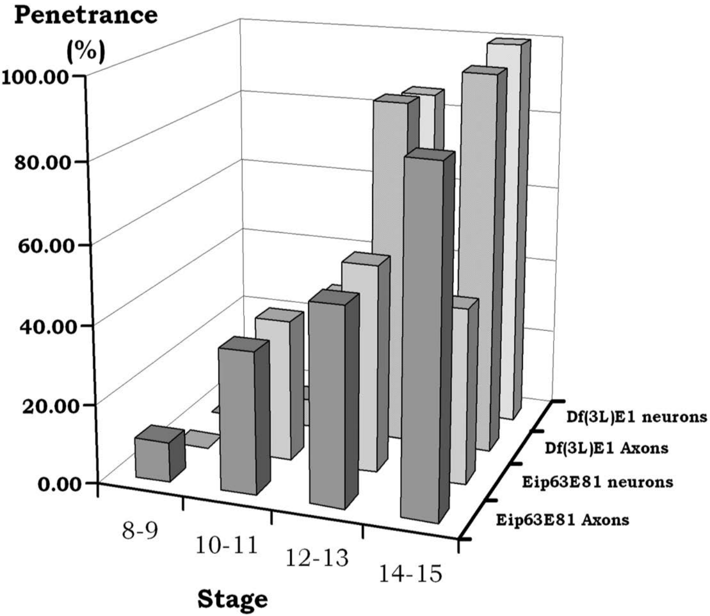

Figure S1

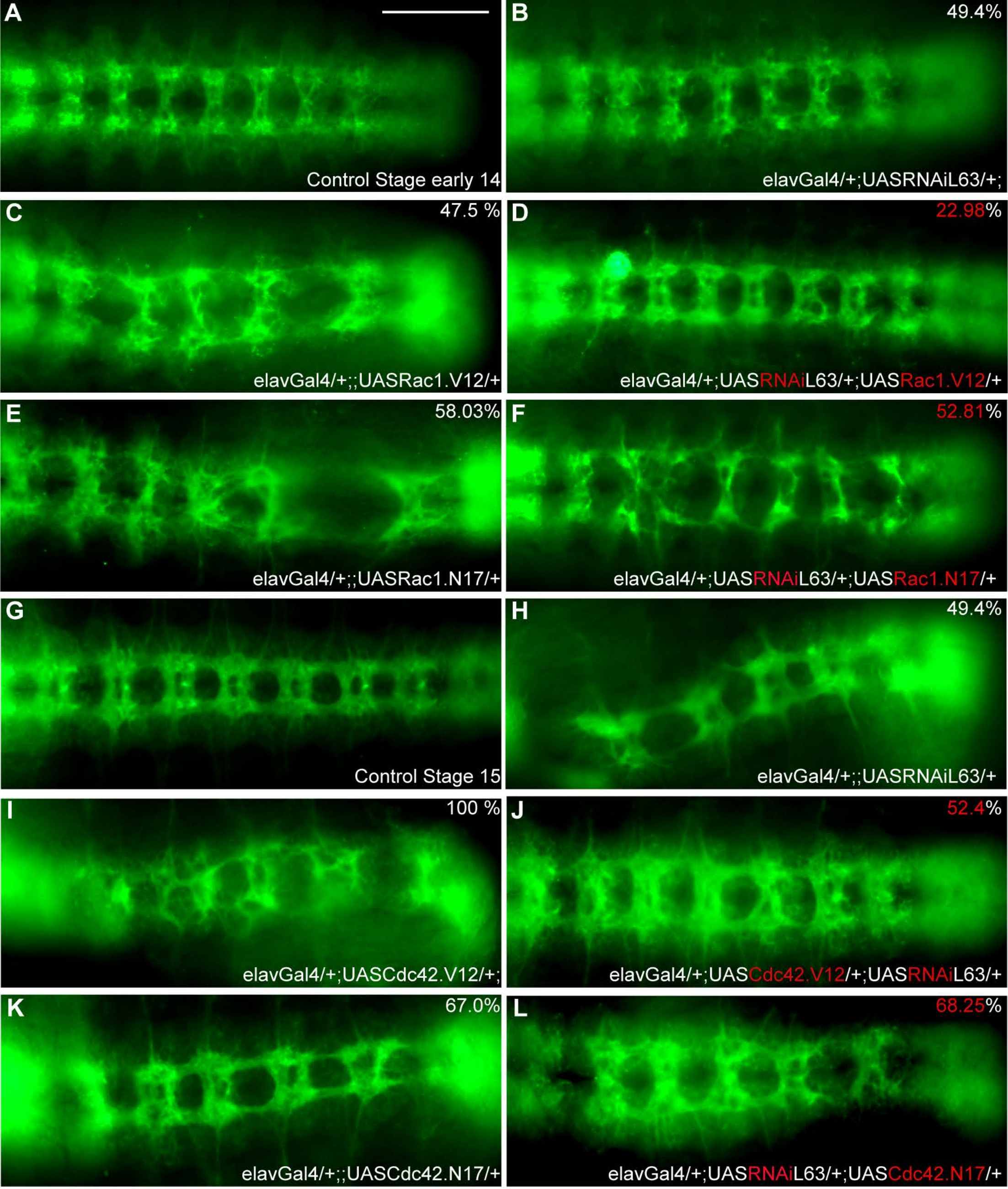
A
Control Stage early 14
B
49.4%
elavGal4/+;UASRNAiL63/+;
C
47.5 %
elavGal4/+;;UASRac1.V12/+
D
22.98%
elavGal4/+;UASRNAiL63/+;UASRac1.V12/+
E
58.03%
elavGal4/+;;UASRac1.N17/+
F
52.81%
elavGal4/+;UASRNAiL63/+;UASRac1.N17/+
G
Control Stage 15
H
49.4%
elavGal4/+;;UASRNAiL63/+
I
100 %
elavGal4/+;UASCdc42.V12/+;
J
52.4%
elavGal4/+;UASCdc42.V12/+;UASRNAiL63/+
K
67.0%
elavGal4/+;;UASCdc42.N17/+
L
68.25%
elavGal4/+;UASRNAiL63/+;UASCdc42.N17/+

Figure S2

A

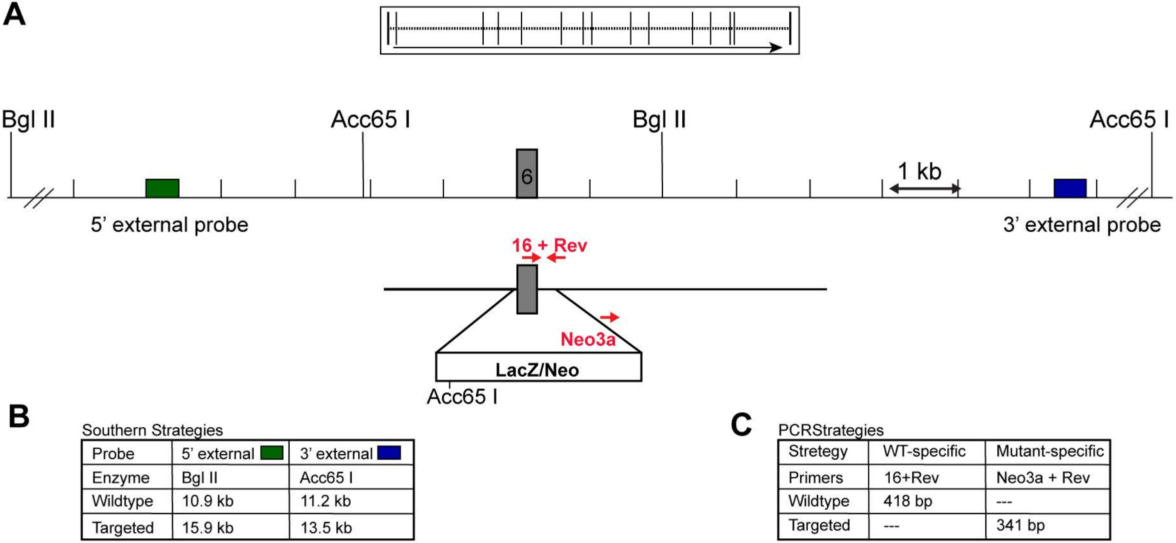

B

Southern Strategies

Probe	5’ external	3’ external
Enzyme	Bgl II	Acc65 I
Wildtype	10.9 kb	11.2 kb
Targeted	15.9 kb	13.5 kb

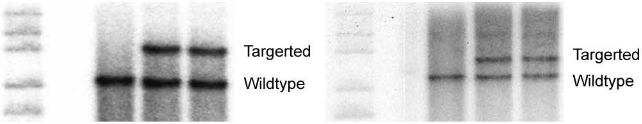

5’ external probe
Bgl II digests
Wildtype 10.9 kb
Targerted 15.9 kb

3’ external probe
Acc65I digests
Wildtype 11.2 kb
Targerted 13.5 kb

* Clone achieving germline transmission

C

PCRStrategies

Stretegy	WT-specific	Mutant-specific
Primers	16+Rev	Neo3a + Rev
Wildtype	418 bp	---
Targeted	---	341 bp

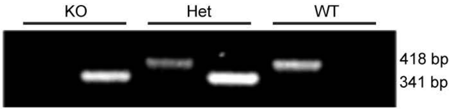

Figure S3

A

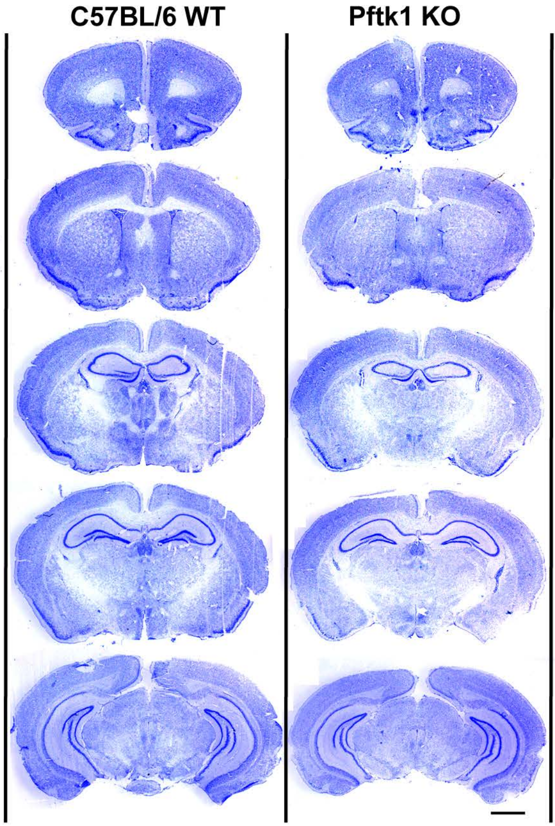

C57BL/6 WT
Pftk1 KO

B

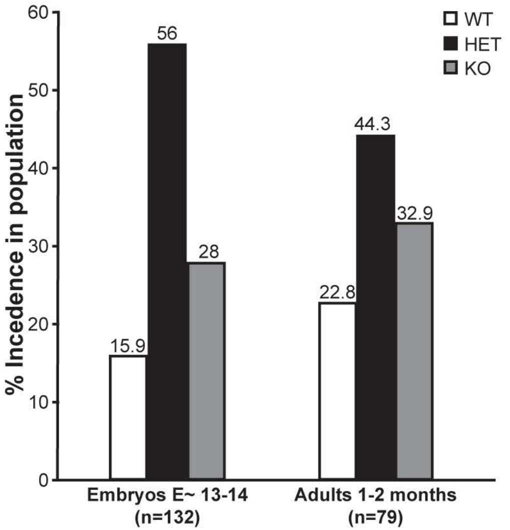

% Incedence in population
60
50
40
30
20
10
0
56
28
15.9
44.3
32.9
22.8
WT
HET
KO
Embryos E~ 13-14
(n=132)
Adults 1-2 months
(n=79)

Figure S4

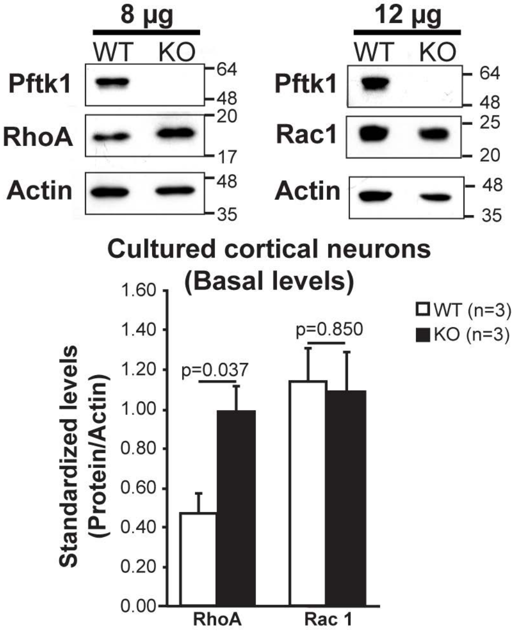
8 µg
WT
KO
Pftk1
RhoA
Actin
64
48
20
17
48
35
12 µg
WT
KO
Pftk1
Rac1
Actin
64
48
25
20
48
35
Cultured cortical neurons
(Basal levels)
Standardized levels
(Protein/Actin)
1.60
1.40
1.20
1.00
0.80
0.60
0.40
0.20
0.00
p=0.037
p=0.850
WT (n=3)
KO (n=3)
RhoA
Rac 1

Figure S5

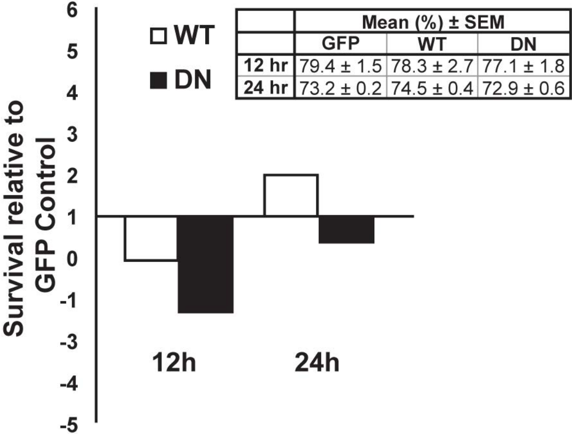

	Mean (%) ± SEM		
	GFP	WT	DN
12 hr	79.4 ± 1.5	78.3 ± 2.7	77.1 ± 1.8
24 hr	73.2 ± 0.2	74.5 ± 0.4	72.9 ± 0.6

Figure S6
