# Supplementary figures and images for: PFTK1 kinase regulates axogenesis during development via RhoA activation
Source: BMC Biol. 2023 Oct 31;21:240. doi: 10.1186/s12915-023-01732-w (PMC10617079; doi:10.1186/s12915-023-01732-w)

Fig 7A

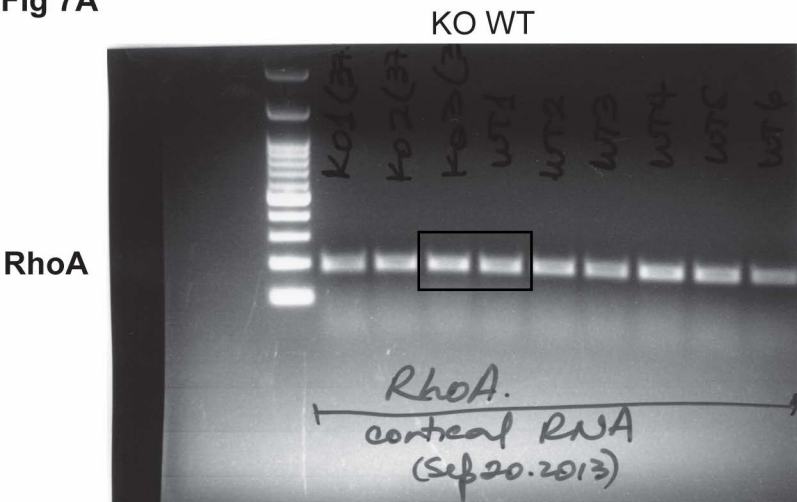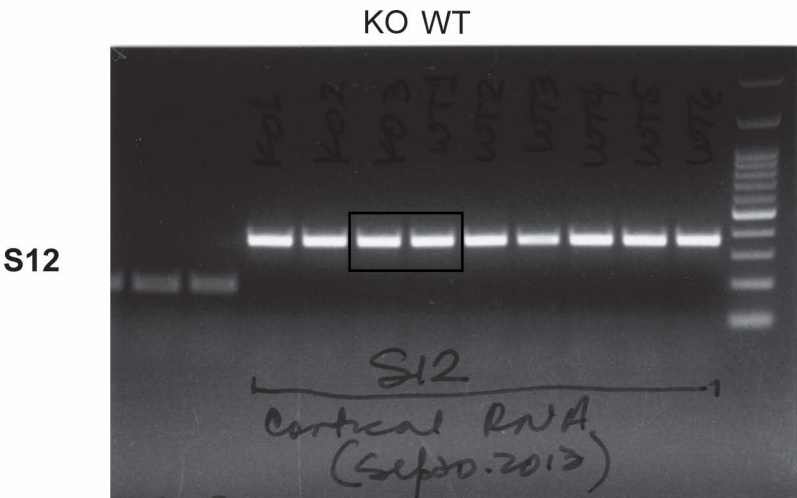

**Fig 7B**

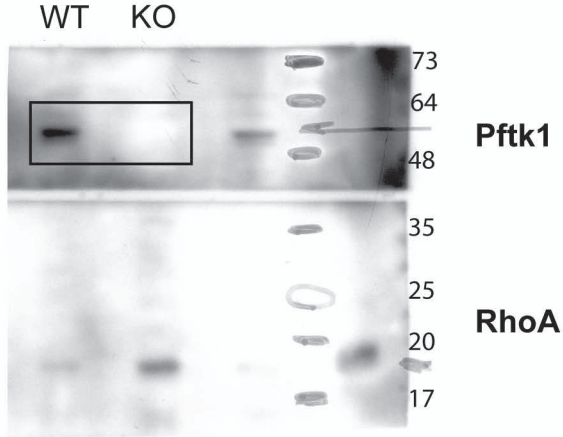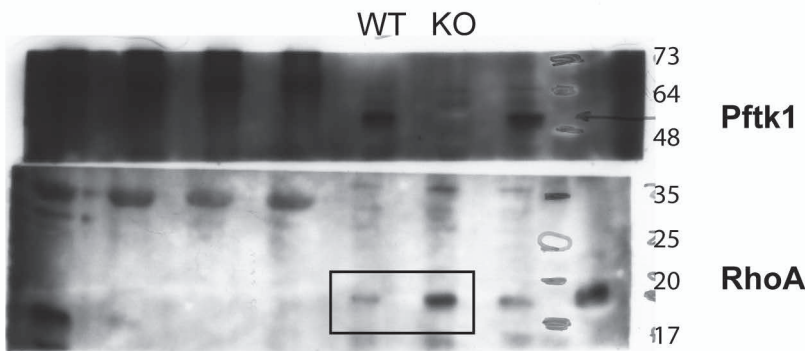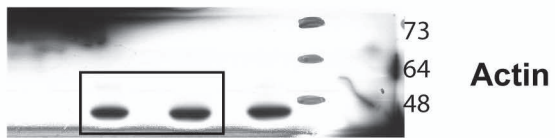

Fig 7D

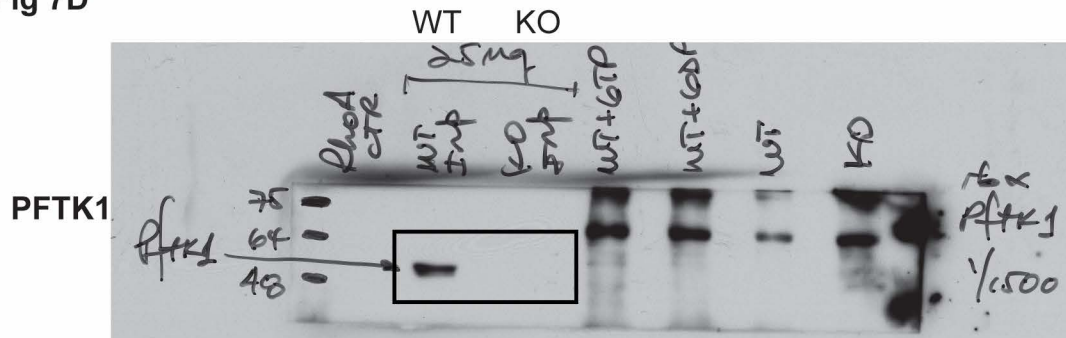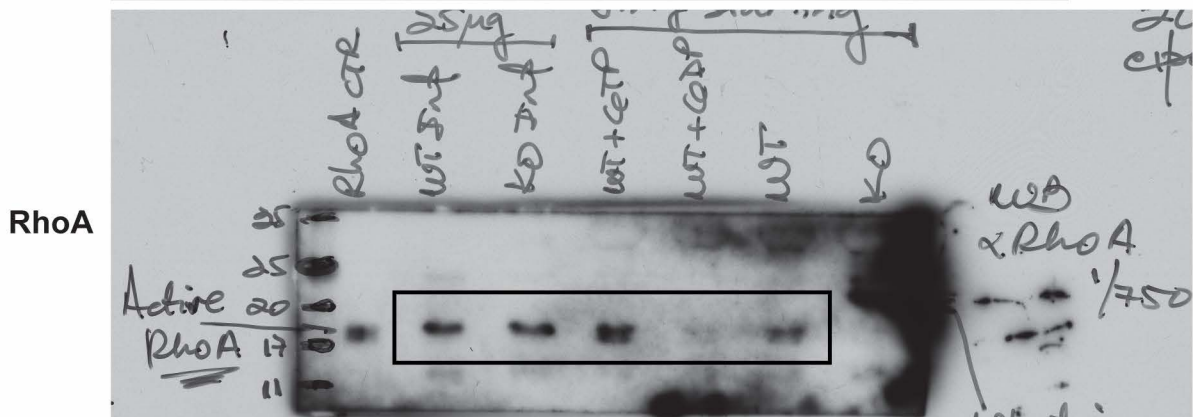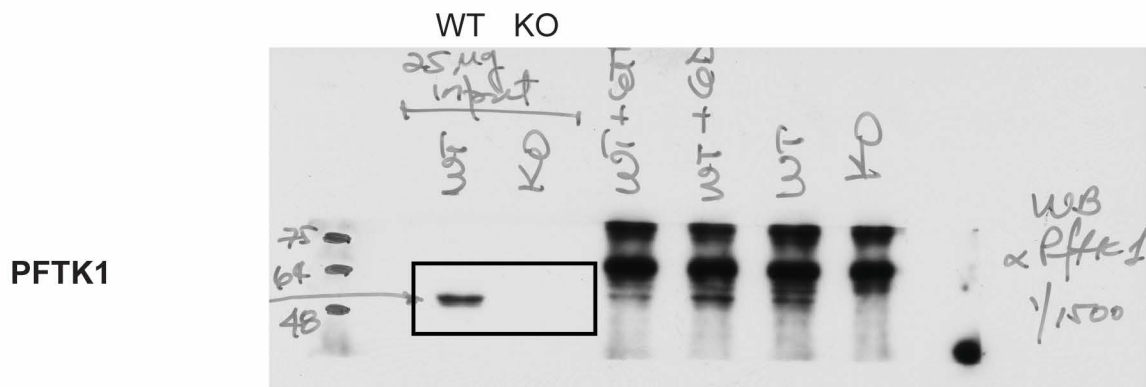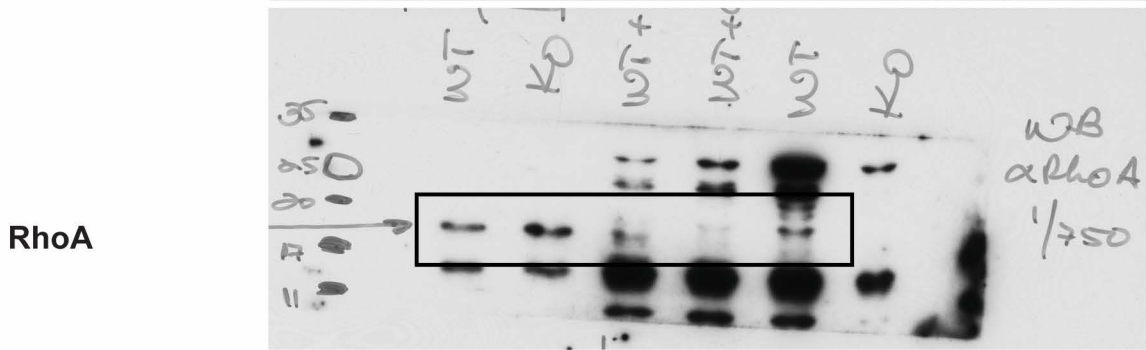

Fig 7E

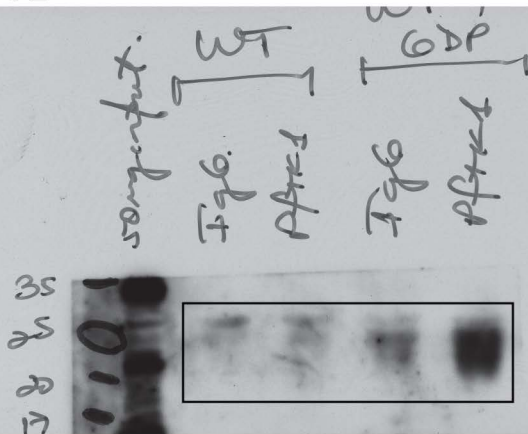

WB  
 $\alpha$  Ser.  
 1/800  
 0/n

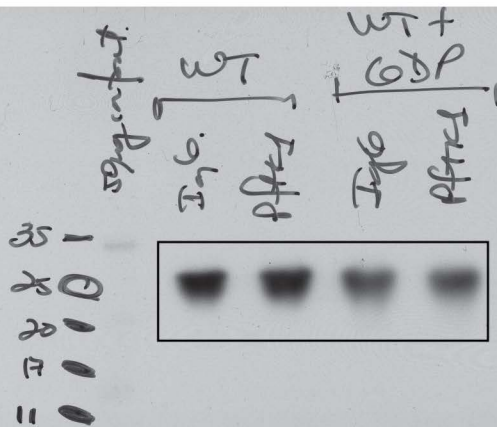

WB.  
 $\alpha$  His  
 1/8,000

Fig S5 8μg

Pftk1

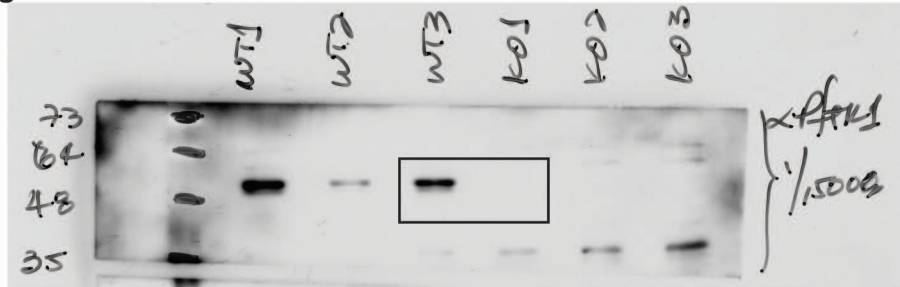

RhoA

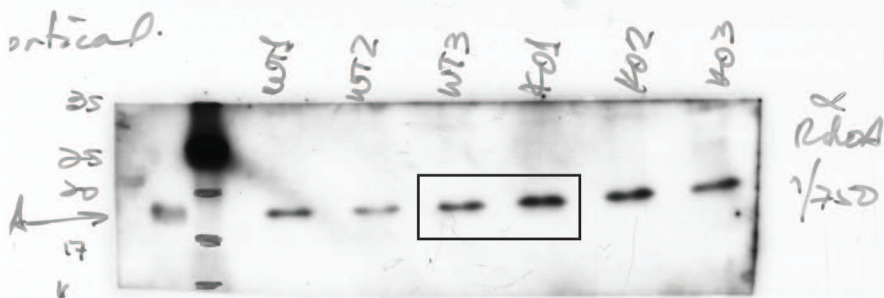

Actin

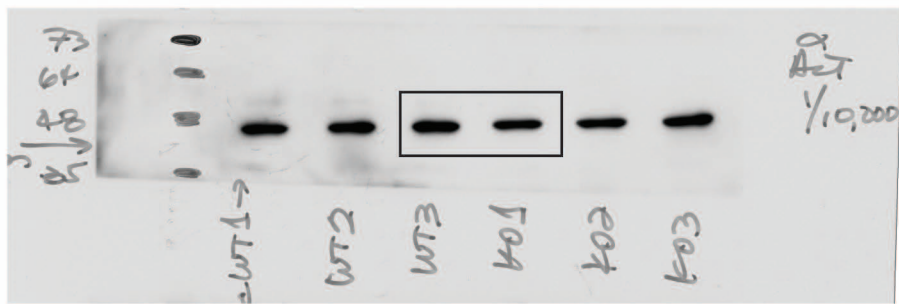

Fig S5 12  $\mu$ g

Pftk1

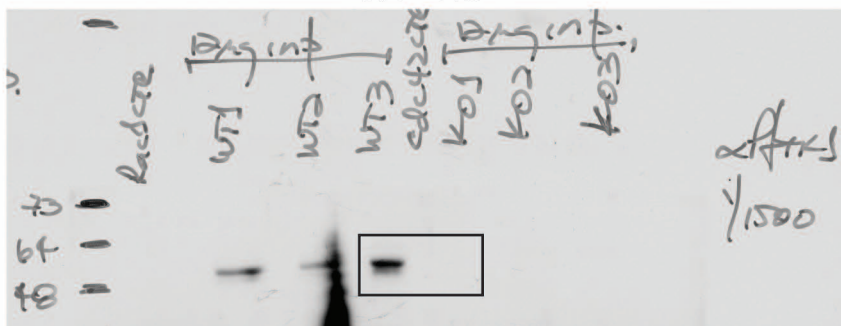

Rac1

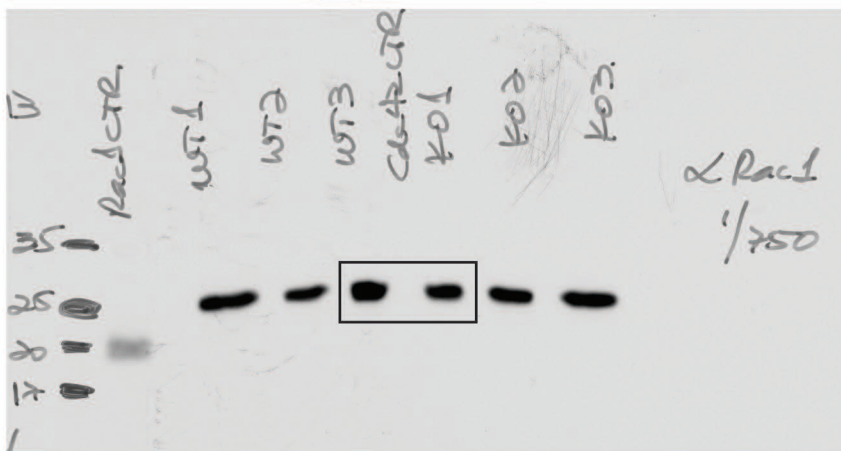

Actin

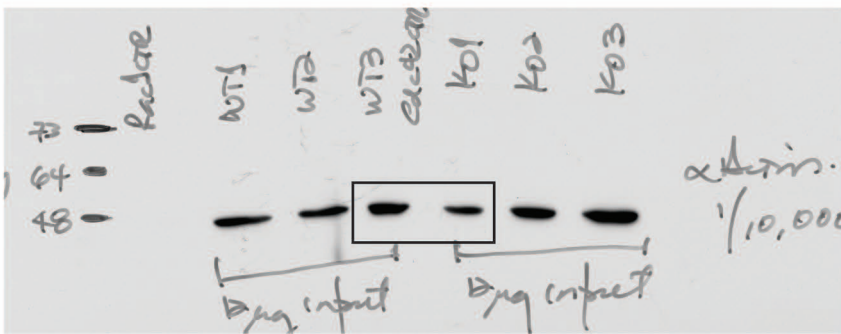

Supplement: Supplementary file 2 — Additional file 2. Uncropped blots. [file 12915_2023_1732_MOESM2_ESM.pdf]
